# Steps to Adapt the Medication Administration Error Survey in Highly Specialised Units—Polish Perspectives

**DOI:** 10.3390/nursrep15050173

**Published:** 2025-05-14

**Authors:** Katarzyna Kwiecień-Jaguś, Wioletta Mędrzycka-Dąbrowska, Monika Kopeć

**Affiliations:** 1 Department of Anaesthesiology Nursing & Intensive Care, Faculty of Health Sciences, Medical University of Gdansk, 80-211 Gdansk, Poland; katarzyna.kwiecien-jagus@gumed.edu.pl; 2 Department of Human Nutrition, University Warmia and Mazury, 10-045 Olsztyn, Poland; monika.kopec@uwm.edu.pl

**Keywords:** medication administration errors, medication safety, patient safety, medication administration error survey, adaptation of an instrument

## Abstract

**Background and Objectives:** Medication errors are a critical issue in healthcare systems worldwide, contributing to significant patient harm, with studies indicating that medication-related incidents are among the most common causes of adverse events in medical practice. There are between 80 and 200 steps in providing a single patient with a single dose of drugs, with five stages, including prescription, preparation, dispensation, administration and monitoring. This study aims to describe and validate the MAEs (Medication Administration Error Scale) tool, which investigates the most common causes of medication errors in medication administration. **Materials and Methods:** Independent translators translated the original version of the scale using language verification. The agreed-upon version of the translation was then assessed by a team of nurses, specialists in anaesthetic and intensive care nursing, in terms of understanding the translated content. After introducing changes resulting from linguistic and organisational differences, a survey questionnaire was prepared and used in the pilot study. Eighty-six respondents participated in the pilot study via the Office 365 platform and the Forms programme. The research was led by nurses who work in highly specialised units. The reliability of the translated version of the questionnaire was examined by calculating the Cronbach’s alpha coefficient. **Results**: The tool’s internal consistency across ranges was within acceptable limits. For part A (questions 1–29), it was 0.93; for part B (questions 30–45), it was 0.94. In part C, regarding the percentages of the type of error occurring in a given medical facility, Cronbach’s alpha coefficient was 0.97. When the factor loadings of the items were evaluated, they were determined to be in the range of 0.602–0.783. In this context, the factor loading levels of the items in the 5-factor model were high and sufficient. **Conclusions:** The statistical analyses suggest that the Polish version of the Medication Administration Error Survey demonstrates satisfactory reliability and is a promising tool for assessing the cause of medication administration errors.

## 1. Introduction

Analysing and reporting errors in drug administration improves the quality of medical services, including systems that strengthen the safety of patients and staff. Reviewing the literature in this area, it is possible to find studies that identify the moment of errors in the medication management process based on analyses of patients’ medical systems/documentation, including medical orders and adverse drug event protocols [1]. Few studies describe research on the voluntary reporting of errors in medication administration, including estimating the frequency of these situations in the clinical environment. The voluntary system for reporting error incidents in drug administration is based on the awareness of medical staff, including doctors, nurses and pharmacists, that errors can occur in clinical work. It is worth monitoring its scale because it improves the safety of both the patient and the staff. A high degree of knowledge among medical workers and an analysis of the factors leading to this event should be essential aspects of managing healthcare facilities. However, this is not a rule and, as scientific research shows, the lack of reporting of events in drug management relates to systemic errors and the lack of appropriate solutions in the hospital environment. This is often associated with the medical staff’s fear of stigmatisation and exclusion of the person who made a mistake and the lack of systemic solutions to improve the quality of staff work or internal procedures [2,3].

Some medical facilities, realising the problem of errors in drug administration, introduce systems that improve the quality of work of medical staff by reducing environmental factors contributing to errors [4]. Among the solutions mentioned are the use of electronic documentation [5], proper labelling of syringes and administered drugs [5], the use of a drug library on infusion pumps [6,7], limiting patient admissions during discharge hours [8], limiting or not allowing telephone consultations during rounds [8], increasing the number of medical staff, the use of Unit Dose pharmacy systems, in which pharmacies prepare drugs for a specific patient [9].

Medication errors have the potential to occur at various stages in the drug administration process, including prescribing, dispensing, preparation, administration and reporting, as highlighted in previous studies and observed in the current study [10]. Medication and medication errors are one of the factors causing unintended harm to hospitalised patients, with approximately 400,000 such cases reported annually in the United States alone [11]. Statistics from the Institute of Medicine show that medication errors account for approximately 19% of all adverse events, leading to 7000 deaths per year [11]. Globally, costs related to medication errors account for approximately 1% of total global healthcare spending. On a worldwide scale, this amount is estimated to be USD 42 trillion [12]. Healthcare costs can be overwhelming, especially with rising expenses. In Poland, more needs to be known about the extent of errors in drug administration and the occurrence of “adverse events” due to medication errors. Due to the wide range of terminology, reporting methods and assessment of complaints handled in different liability modes, it is difficult to determine the number of error cases. Professional Responsibility Ombudsmen operate within medical professional associations and courts and have detailed insight into these issues. However, these data are not readily available to the public [13].

The issue of errors in the medication administration process can impact any employee involved in the process, with the nursing staff primarily responsible for medication preparation. Emphasising patient safety in educating nursing personnel regarding the preparation and administration of medicinal products, the ten safety rules (10 “R”—10 Rights Rule in Drug Administration) guide nursing staff in this process. These rules encompass ensuring the right patient, the correct dose, the right administration time, the proper route of administration, documentation of the administered products and monitoring for side effects. Despite the knowledge of these rules, errors in drug administration persist. The 10 “R” rule holds significant importance, advocating the understanding of the causes of medication errors and implementing strategies to reduce such errors, ensuring safe practice throughout the treatment process, from chemical preparation to monitoring drug side effects and interactions [14].

The measurement of the magnitude of MAE is challenging because of the limited tools available. The community and patients expect safety standards and systems to support clinical practice. One of the methods to detect MAE is direct observation. That approach can be used among newly graduated nurses. To more experienced staff, a questionnaire and self-reflection give more open and formative feedback [15]. One of those tools is the Medication Administration Survey (MAE) prepared by Professor Wakefield. The scale was used in many scientific projects; its reliability was calculated based on alfa Cronbach index. The scale investigates the most common causes of medication administration error and helps estimate the scale of the problem. The tool was used and validated for the first time in 1994. Since then, the tool has been updated and implemented in different countries, providing good feedback to the unit and hospital managers. This was one of the reasons why this scale was chosen to implement in the Polish environment. The second rationale was the changes in Polish law regulation [16,17,18,19,20,21]. According to those changes, collecting information about the challenges associated with the drug administration process, potential risks, and the scale of the problem is also crucial. In 2023, a new regulation was introduced in Poland, which, immediately after being imposed on hospitals, introduced systems for monitoring health errors, including pharmacotherapy errors [22]. Medical facilities are obligated to monitor those adverse situations and implement corrective actions. New regulations provide a new approach to the accreditation process for hospitals and medical facilities and establish new standards.

By assessing the extent of the problem and the factors that disrupt the preparation of medical products, we can develop systems to prevent such situations. Since nursing staff play a critical role in preparing and administering medications, it is essential to understand their perspectives on error reporting in the hospital environment, identify potential solutions to improve the situation and determine the scale of the problem.

### Aim of the Study

This study aims to describe and validate the MAEs (Medication Administration Error) tool, which investigates the most common causes of medication errors in the medication administration process. The tool is also used to estimate the number of injection and non-injection drug errors in the hospital environment. The manuscript demonstrates the initial process of adapting the scale to Polish conditions. This study is the first in Poland to focus on validating a tool among anaesthesiology and intensive care nurses. Previous studies on the safety of pharmacotherapy have mainly focused on drug pharmacokinetics, side effects and the role of clinical pharmacotherapy [23]. The first Polish study on medication errors was published in 2022. Still, the research was conducted based on a questionnaire prepared for the study “Attitudes and beliefs of health services about the causes and reporting of treatment errors in the British intensive care unit” [24,25]. There have been no studies analysing the scale of the problem through the identification and reporting system on medication administration errors. That is why it is crucial to know the scale of the problem, as well as the attitude and perception of the medical staff regarding errors in drug administration.

## 2. Materials and Methods

### 2.1. The Scale Was Adopted Due to the Polish Environment, as Outlined in the Following Steps from the Literature [26]

#### 2.1.1. Step 1—Cross-Cultural Adaptation of the Scale

The first step was focused on the cross-cultural adaptation of the existing scale using the translation method. Experts in cross-cultural studies highly recommend that method. The process was carried out as follows:

- The translation of the tool;
- Evaluation by the Review Committee;
- Resending of the information about changes in the Polish version of the scale to the first author;
- Conducting a pilot study;
- Carrying out a descriptive and reliability analysis using statistical data.

#### 2.1.2. Step 2—Psychometric Validation of Translated Scale

To evaluate the psychometric properties of the Polish Version of the Medication Administration Errors Scale, multi-factor analysis was used. The most common application for factor analysis is to reduce the large number of variables to fewer or to test the relationship between concepts [27,28].

### 2.2. Medication Administration Survey (MAEs)

The literature shows that errors in the area of drug administration are present in the clinical environment and have a very complex nature. Some medical facilities, aware of the scale of the problem, monitor the number of such events or situations in which an employee is close to making a mistake. Analysing the scale of the risk of errors in drug administration limits the effects of this type of event. It is essential in developing IT systems and solutions to prevent these errors. This challenge was taken up by Prof. Bonnie Wakefield, who, together with her team, developed a scale to understand the causes of errors in the medication administration process. The instrument was created by a clinically experienced physician involved in quality improvement in healthcare. Based on contemporary clinical trials and the investigator’s experience, individual statements were developed to reflect the most common causes of medication administration errors and why such a low percentage of errors are reported. The developed MAE survey was consulted with a team of experienced nursing staff, who provided their suggestions.

Due to this, the tool was improved, and a pilot study was conducted in selected medical facilities in 1994. In 1996, the survey questionnaire was updated, considering community feedback and new scientific evidence in this area [16].

Completing the survey questionnaire takes no more than 10 min. The questionnaire consists of three parts. Part one A includes questions about the reasons for errors in medication administration. In the initial version, this part of the survey questionnaire included 19 questions, and was later expanded to 29. The second part of the questionnaire, B, contains questions about the reasons for not reporting errors. The last part of the tool—C—allows the respondent to determine, based on their clinical experience, the percentage of medical errors occurring when administering injectable and non-injectable drugs in their facility [16].

In parts A and B, respondents choose the appropriate answer for each statement using a 6-point Likert scale, where the answer “I strongly disagree” is assigned 1 point, and “I strongly agree” is assigned 6 points. In part C, in which the respondent selects the percentage of errors in the field of injectable and non-injectable drugs on a scale, each value was assigned several points on a 10-point scale. For responses 0–20%, the point value is 1. Respondents are also asked to indicate the percentage of errors in injectable and non-injectable drugs reported in their workplace. The last part of the questionnaire is a summary containing socio-demographic questions relating to age, gender, position and the number of transfers to other hospital wards [16].

### 2.3. Practical Use of the Medicines Administration Errors (MAEs) Questionnaire

The usefulness of the tool developed by Prof. B. Wakefield and its reliability, calculated based on Cronbach’s alpha index, were checked in various scientific projects starting in 1994 [17,18]. Currently, the MAE’s questionnaire is still successfully used in scientific projects in both conservative and surgical departments [19,20,21].

### 2.4. The Medication Administration Error Reporting Survey in Poland

Work on validating and adapting the Polish version of the MAE’s scale was initiated at the Department of Anaesthesiology and Intensive Care Nursing, Medical University of Gdańsk. Before starting the work, the consent of the scale’s author, Prof. Bonnie Wakefield, was obtained, who provided enormous substantive support in using the scale for scientific research. In adapting the scale, it was necessary to take into account certain linguistic and cultural differences as well as organisational differences in hospitals.

Adapting the tool to Polish conditions is an extremely difficult task. It requires considering specific differences in the target population’s understanding of the concept itself. Some difficulties were also related to the lack of equal standards for drug administration in certain medical units and hospitals.

An independent translation agency translated the original version of the Medication Administration Error Reporting Survey scale and proofread it by a native speaker. Then, the translated survey questionnaire was distributed to a group of nurses. The experts were asked to evaluate items of the scale and the overall structure of the questionnaire. The team analysed the tool and assessed the translated scale for clarity and understanding of the content of the questions. The panel experts had not only a clinical but also an academic background. The criteria to choose the panel experts were healthcare professionals who were registered in the Nursing Chamber and had at least 5 years of clinical experience in highly specialised units like intensive care, cardio ICU and anaesthesia, or agreed to participate in the panel. After discussing the translated version of the questionnaire, 5 expert group members shared their opinions and suggestions with the main researcher. The evaluation process showed that in Part A, questions 25 and 26 raised the most doubts. Question 25 in the original translation was, “Medicine orders are incorrectly entered into Kardex”. Question 26 is also related to the Kardex IT system. In Poland, the Regulation of the Minister of Health from 6 April 2020 specifies that patient medical records should be kept within the ICT system. There are no recommendations regarding the type of system used. Therefore, each medical facility has the right to choose software. Of course, the documentation template should meet the general conditions regarding the data that medical documentation must contain [28,29,30,31]. For this reason, the name “Kardex” was changed to the word “electronic/paper order system”. The scale author was informed about the need to introduce such a change and approved it. The new, up-to-date version of the questionnaire was again presented to the panel of the same experts. They all approved the changes. The pilot study was conducted from March 2023 to the end of June 2023 after obtaining the consent of the Bioethics Committee of the Medical University of Gdańsk (NKBBN/185/2023). The Supplementary Material in Tables S1–S3 presents an example of the MAE tool’s translated version.

The pilot study examined whether the tool helps assess the most common causes of medication errors and the scale of the problem. The research was directed to the staff of highly specialised units, like anaesthesiology and intensive care departments. The questionnaire was prepared on the Office 365 platform in the Forms programme. The link to the survey questionnaire was sent to the anaesthesia and intensive care nurse forums with the consent of the platform administrators. Before starting the study, each participant had to familiarise themselves with the purpose of the research and express their informed consent to participate in the project by checking the “yes” answer to the statement “I consent to participate in the study, also declaring that I am an adult”. Failure to select an affirmative answer resulted in the inability to complete the questionnaire. The acquired anonymised data were saved in the cloud and password-protected. Due to technical reasons, the reasons for the lack of consent to participate in the study were not investigated.

## 3. Results

### 3.1. Step 1—Descriptive Analysis with Cronbach’s Alpha Calculation

A total of 86 respondents took part in the pilot study. Analysing the age and sex structure of the respondents, most of the nurses were female (87.21%; n = 75), and their ages ranged from 31 to 50 years (56.98%, n = 49). More than 67% of respondents had bachelor’s degrees in nursing (67.34%, n = 58) and had specialisation in anaesthesiology and ICU (87.21%; n = 75). The respondents were employed in different reference hospital levels. The detailed characteristics of the nurses are presented in Table 1.

The internal consistency of the tool was assessed using Cronbach’s alpha coefficient, which can range between 0 and 1. The interpretation of Cronbach’s alpha values is as follows: >0.90 is considered very good/excellent; between 0.80 and 0.90 is good; between 0.70 and 0.80 is acceptable; between 0.60 and 0.70 is weak; and <0.60 is unacceptable [32]. The internal consistency of the tool across ranges was within acceptable limits. For part A (questions 1–29), it was 0.93, for part B (questions 30–45), 0.94. In the case of part C, the percentage of errors occurring in a given medical facility was 0.97 (Table 2).

### 3.2. Step 2—Exploratory Factor Analysis

Multifactorial analysis was used to validate the changed version of the scale. The data obtained allowed for assessing the properties and structure of the individual subscales used in the questionnaire. The Kaiser statistic considers the calculations regarding sample adequacy and the variance index between variables, indicating that the sample was adequate for Exploratory Factor Analysis [26,27,28].

The scree plot and the Cattell point are tools used to reduce the number of variables to the main factors. We used a scree plot with the Cattell point to identify the primary components. Selecting the number of primary components involves locating the point on the graph where the slope begins to flatten out. In simpler terms, this is the point where the graph stops “crumbling”. Any points above this scree point indicate the number of main components that can be extracted for further analysis. Based on the scree plot and the criterion of a value greater than 2.0 (with a total percent variance of 47.21%), we identified five main components for extraction (see Figure S1). The cumulative variance rates explained by the factors were 25.903% for Factor I, 40.187% for Factor II, 48.667% for Factor III, 54.020% for Factor IV and 58.012% for Factor V (Table 3). The rate of variance explained by the assessment tool should be at least 40%. In this context, the scale has an explained total variance rate above the lowest explained total variance rate reported in the literature.

In the literature, factor load values of ≥0.45 for items have been reported to be sufficient criteria for item selection. When the factor loadings of the items were evaluated, they were determined to be in the range of 0.602–0.783. In this context, the factor loading levels of the items in the 5-factor model were high and sufficient.

## 4. Discussion

### 4.1. Scale Validity and Reliability

This study aimed to translate, adapt and validate the Polish version of the Medication Administration Error Questionnaire (MAE) specifically for highly specialised nurses. The translation and validation of the MAE scale were carried out to reflect Poland’s unique cultural and hospital environmental context.

Experts in the field reviewed and approved the translation process, and it was decided that two questions from the original version required modification. Nurse specialists with university and clinical experience recommended changes to two questions in Part A of the tool. Specifically, Question 25 in the original wording stated, “Medicine orders are incorrectly entered into Kardex”. Question 26 also referred to the Kardex IT system. Since there are no standardised recommendations in Poland concerning the IT systems used for order management, each medical facility can select its own software. Consequently, “Kardex” was replaced with “electronic/paper order system” to reflect this flexibility [29,30,31]. The researcher communicated this change to the author of the scale, and Professor Wakefield approved it. After implementing these revisions, the researchers proceeded to the next phase of the validation process. The internal consistency of the questionnaire was assessed using Cronbach’s alpha coefficient, which ranges from 0 to 1. The interpretation of Cronbach’s alpha values is as follows: greater than 0.90 is considered very good/excellent; between 0.80 and 0.90 is categorised as good; from 0.70 to 0.80 is deemed acceptable; between 0.60 and 0.70 is considered weak; and below 0.60 is labelled unacceptable [32]. The results indicated that Cronbach’s alpha for Parts A, B and C exceeded 0.90, marking an excellent outcome. This result, obtained through statistical analysis, was satisfactory and closely aligned with other authors’ reports—the final step in the validation process involved conducting a multifactorial analysis [16,17,18,19,20]. The results revealed high and significant factor loadings. However, comparing these findings with existing analyses is challenging since prior studies have predominantly focused on calculating Cronbach’s alpha for individual questionnaire subscales.

Overall, the Polish version of the MAE questionnaire has been successfully adapted and validated, offering a reliable tool tailored to the local context of medication administration among nurses in Poland.

### 4.2. Contribution to the Nursing Field in High Specialistic Units

Errors in the area of drug administration may occur at any stage of the process, leading to negative effects on health and, in extreme cases, the death of the patient. Some studies show that highly specialised departments, such as anaesthesiology or intensive care, are more exposed to this event [33]. According to data, the incidence of errors in the area of drug administration in intensive care ranges from 1.2 to 947 errors per 1000 days of hospitalisation [34]. In the case of anaesthesiology and the operating theatre, errors have been determined to be between 7.3 and 12% [30,31]. This increased risk is due to many factors. Patients in intensive care units are often patients with failure of one or more systems/organs, which require specialised medications, including high-risk medicines, which, if misused, carry the risk of causing harm to the patient [35]. When discussing anaesthesiology and the operating theatre, one should remember the need to work under tremendous time pressure, where clinical decisions must be made vigorously, considering the anaesthetised patient’s current condition [36,37]. Anaesthesiology represents a rapidly evolving medical speciality covering peri-operative, pre-hospital and in-hospital critical emergency management, intensive care medicine and pain management. In Poland, the development of intensive care units has been closely connected to the development and advances of anaesthesiology. Although in some countries, intensive care units may be run and managed by other medical specialists like surgeons, in Poland, for many years, the anaesthesiologist and the anaesthesiology and ICU nurse have been the main pillars of those units, cooperating with other specialists. For this reason, in Poland, these two broad branches of medicine function inseparably as anaesthesiology and intensive care medicine [38].

Collecting information on errors in the area of drugs used makes it possible to detect the mistakes, estimate the number of such situations in the hospital environment, monitor changes introduced in the system and assess its efficiency. Analyses of available literature on the subject show that several solutions allow for data collection on errors in administered medications. Despite central systems in most European countries, many medical facilities use voluntary and anonymous reporting systems [39]. Voluntary reporting of incidents related to errors in the area of medication administration is based not only on staff awareness of the nature of errors in the area of medication administration but also on recognition, reporting, preparation of a report and implementation of corrective actions. Although this four-step process is relatively simple, several factors can make reporting difficult, such as management practices, the culture of work organisation and punishing the individual even when the error appears due to the lack of solutions [16]. A good example of monitoring adverse events and errors may be Switzerland’s instructive example. In 1996, a voluntary programme was initiated to record the most common adverse events and errors. Reports in paper or electronic form concern the 50 common medical interventions in internal medicine, including pharmacotherapy. The data obtained in this way is published in the Foundation for the Prevention of Complications in Internal Medicine bulletin. Over 10 years, reports were received from 32 clinics regarding over 250,000 interventions and nearly 9000 adverse events (3.6%) [40].

The system for reporting adverse events and medication errors is crucial for Polish medical facilities. In light of the new legal regulations introduced in 2023, hospital managers must establish an internal programme to monitor so-called adverse events and ensure service quality. It is essential to accurately identify the factors contributing to the event of medication errors and modify them at the site of occurrence. Before the implementation of the new regulation, the previous Central System of Adverse Events did not provide adequate data regarding the scope of the issue. The regulations enacted in 2023 are somewhat innovative and are expected to overhaul the medical facilities’ accreditation process and funding. Under the new law, managers are tasked with educating staff about the scale of the problem and monitoring and implementing solutions to identify and mitigate the risks that lead to these situations. Failure to comply with the new organisational requirements may result in financial penalties for the facility and restrict its ability to carry out medical activities [14].

The validation project showed that the Polish adaptation of the MAE scale had satisfactory measurement properties and can be used in highly specialised departments to evaluate the factors that lead to medication error and monitor the scale of the problem.

## 5. Conclusions

The Polish version of the MAE tool demonstrates promising reliability for assessing the cause of medication administration errors in high-intensity departments like intensive care units and anaesthesiology. However, further validation in larger, more diverse samples is necessary to confirm its broader applicability. The survey questionnaire effectively provides employees with the opportunity to report the causes of errors in medication administration confidentially, identify factors contributing to the occurrence of errors, continuously monitor safety improvements and assess the patient safety culture in terms of the effectiveness of solutions implemented in the facility.

## 6. Limitations of the Study

A limitation of this research was the study group’s selection and size. The number of respondents does not fully represent the broader nursing community in Poland. In the future, the authors plan to lead a project that will involve a more diverse and more extensive sample of respondents. The pilot study showed the satisfactory reliability of the Polish version of the Medication Administration Error survey, which can be used in the future, as it is a good tool for estimating the reasons for medication errors, reasons for non-reported MAEs and the percentage of such incidents in clinical settings.

## Figures and Tables

**Table 1:** Sociodemographic characteristics.

Age	N	%
<30 years old	15	17.44
31–50 years old	49	56.98
more than 50	22	25.58
**Sex**		
F	75	87.21
M	11	12.79
**Nursing Education**		
Bachelor in Nursing	58	67.44
Master in Nursing	24	27.91
Registered Nurse	4	4.65
**Additional qualifications, courses**		
Specializtion	75	87.21
Qualification course	11	12.79
**Years of experience**		
5–10> years	11	12.79
10–20> years	25	29.06
20–30>years	13	15.12
30–40 years	10	11.63
**Current position**		
Staff Nurse	61	70.93
Head Nurse	17	19.77
Contract Nurse	8	9.30
**Type of employment**		
Full-time	71	82.56
Part-time	6	6.98
Contract	9	10.46
**Hospital level due to the European Reference Hospital Network**		
III	30	34.88
II	34	39.53

**Table 2:** Reliability analysis of the Medication Administration Errors Scale (MAE tool).

Reliability Measure: Cronbach’s Alpha	Value	Interpretation
Part A of the questionnaire (questions 1–29)	0.93	very good
Part B of the questionnaire (questions 30–45)	0.94	very good
Part C of the questionnaire	0.97	very good

**Table 3:** Results of exploratory factor analysis (n = 86).

	Subscales Items	Factor Load Value	Eigenvalue	Variance (%)	Cumulative Variance (%)
Factor I	1. The names of many medications are similar.	−0.560	16.837	25.903	25.903
	4. Physicians’ medication orders are not legible.	−0.523			
	5. Physicians’ medication orders are not clear.	−0.605			
	6. Physicians change orders frequently.	−0.637			
	7. Abbreviations are used instead of writing the orders out completely.	−0.618			
	34. Medication error is not clearly defined.	−0.568			
	35. Nurses may not think the error is important enough to be reported.	−0.610			
	36. Nurses believe that other nurses will think they are incompetent if they make medication errors.	−0.720			
	37. The patient or family might develop a negative attitude toward the nurse, or may sue the nurse if a medication error is reported.	−0.663			
	38. The expectation that medications be given precisely as ordered is unrealistic.	−0.571			
	39. Nurses are afraid the physician will reprimand them for the medication error.	−0.590			
	40. Nurses fear adverse consequences from reporting medication errors.	−0.643			
	41. The response by nursing administration does not match the severity of the error.	−0.662			
	42. Nurses could be blamed if something happens to the patient as a result of the medication error.	−0.605			
	43. No positive feedback is given for passing medications correctly.	−0.680			
	44. Too much emphasis is placed on med errors as a measure of the quality of nursing care provided.	−0.551			
	45. When med errors occur, nursing administration focuses on the individual rather than looking at the systems as a potential cause of the error.	−0.557			
	64. Wrong fluid	−0.562			
	65. Wrong rate of administration	−0.538			
Factor II	48. Wrong patient	0.650	9.285	14.285	40.187
	49. Wrong dose	0.621			
	50. Wrong drug	0.726			
	51. Medication is omitted	0.695			
	52. Medication is given, but has not been ordered by the physician	0.560			
	53. Medication administered after the order to discontinue has been written	0.561			
	55. Wrong method of administration	0.554			
	57. Wrong patient	0.731			
	58. Wrong dose	0.719			
	59. Wrong drug	0.679			
	60. Medication is omitted.	0.640			
	61. Medication is given, but has not been ordered by the physician.	0.612			
	62. Medication administered after the order to discontinue has been written	0.623			
	63. Given to patient with a known allergy	0.642			
Factor III	26. Errors are made in the electronic/paper order system	−0.519	5.512	8.480	48.667
	2. Different medications look alike.	0.588			
	3. The packaging of many medications is similar.	0.612			
Factor IV	9. Pharmacy delivers incorrect doses to this unit.	−0.503	3.480	5.353	54.020
	10. Pharmacy does not prepare the med correctly.	−0.525			
	11. Pharmacy does not label the med correctly.	−0.522			
Factor V	18. Nurses on this unit have limited knowledge about medications.	0.547	2.594	3.991	58.012

## Data Availability

The data that support the findings of this study are available from the corresponding author upon reasonable request.

## Supplementary Materials

The following supporting information can be downloaded at https://www.mdpi.com/article/10.3390/nursrep15050173/s1. Table S1. The Medication Administration Error Scale (MAE)—part A. Table S2. The Medication Administration Error Scale (MAE)—part B. Table S3. The Medication Administration Error Scale (MAE)—part C. Figure S1. Scree plot.

## Public Involvement Statement

There was no public involvement in any aspect of this research.

## Guidelines and Standards Statement

This manuscript was drafted against the STROBE Statement for cross-sectional studies.

## References

[1] Yoon S., Sohng K. (2021). Factors causing medication errors in an electronic reporting system. Nurs. Open.

[2] Brabcová I., Hajduchová H., Tóthová V., Chloubová I., Červený M., Prokešová R., Malý J., Vlček J., Doseděl M., Malá-Ládová K. (2023). Reasons for medication administration errors, barriers to reporting them and the number of reported medication administration errors from the perspective of nurses: A cross-sectional survey. Nurse Educ. Pract..

[3] Aljabari S., Kadhim Z. (2021). Common Barriers to Reporting Medical Errors. ScientificWorldJournal.

[4] Ciapponi A., Fernandez Nievas S.E., Seijo M., Rodríguez M.B., Vietto V., García-Perdomo H.A., Virgilio S., Fajreldines A.V., Tost J., Rose C.J. Reducing Medication Errors for Adults in Hospital Settings. Cochrane Effective Practice and Organisation of Care Group, Editor. Cochrane Database Syst Rev [Internet]. http://doi.wiley.com/10.1002/14651858.CD009985.pub2.

[5] Aceves-Gonzalez C., Caro-Rojas A., Rey-Galindo J.A., Aristizabal-Ruiz L., Hernández-Cruz K. (2024). Estimating the impact of label design on reducing the risk of medication errors by applying HEART in drug administration. Eur. J. Clin. Pharmacol..

[6] Elliott R.A., Angley M., Criddle D.T., Emadi F., Liu S., Penm J. (2024). Achieving safe medication management during transitions of care from hospital: Time for a stewardship approach. Aust. Prescr..

[7] Wang Y., Zhang X., Hu X., Sun X., Wang Y., Huang K., Sun S., Lv X., Xie X. (2022). Evaluation of medication risk at the transition of care: A cross-sectional study of patients from the ICU to the non-ICU setting. BMJ Open.

[8] Manias E., Street M., Lowe G., Low J.K., Gray K., Botti M. (2021). Associations of person-related, environment-related and communication-related factors on medication errors in public and private hospitals: A retrospective clinical audit. BMC Health Serv. Res..

[9] Tu H.N., Shan T.H., Wu Y.C., Shen P.H., Wu T.Y., Lin W.L., Yang-Kao Y.H., Cheng C.L. (2023). Reducing Medication Errors by Adopting Automatic Dispensing Cabinets in Critical Care Units. J. Med. Syst..

[10] Tabatabaee S.S., Ghavami V., Javan-Noughabi J., Kakemam E. (2022). Occurrence and types of medication error and its associated factors in a reference teaching hospital in northeastern Iran: A retrospective study of medical records. BMC Health Serv. Res..

[11] Latif A., Kruer R., Jarrell A. (2014). Reducing medication errors in critical care: A multimodal approach. Clin. Pharmacol. Adv. Appl..

[12] WHO Medication Without Harm [Internet]. https://www.who.int/initiatives/medication-without-harm.

[13] IPSOS Reduction Errors in Pharmacotherapy in Poland [Internet]. https://ecamet.eu/wp-content/uploads/2022/03/21016698-IN0107-EAASM-Medication-errors_Poland.pdf.

[14] Edwards S., Axe S. (2015). The 10 ‘R’s of safe multidisciplinary drug administration. Nurse Prescr..

[15] Mukhalalati B.A., Taylor A. (2019). Adult Learning Theories in Context: A Quick Guide for Healthcare Professional Educators. J. Med. Educ. Curric. Dev..

[16] Wakefield B.J., Uden-Holman T., Wakefield D.S. (2005). Development and Validation of the Medication Administration Error Reporting Survey. Advances in Patient Safety: From Research to Implementation [Internet].

[17] Wakefield B.J., Wakefield D.S., Uden-Holman T., Blegen M.A. (1998). Nurses’ perceptions of why medication administration errors occur. Medsurg Nurs..

[18] Wakefield D.S., Wakefield B.J., Uden-Holman T., Borders T., Blegen M., Vaughn T. (1999). Understanding Why Medication Administration Errors May Not Be Reported. Am. J. Med. Qual..

[19] Alzoubi M.M., Al-Mahasneh A., Al-Mugheed K., Al Barmawi M., Alsenany S.A., Farghaly Abdelaliem S.M. (2023). Medication Administration Error Perceptions Among Critical Care Nurses: A Cross-Sectional, Descriptive Study. J. Multidiscip. Healthc..

[20] Bifftu B.B., Dachew B.A., Tiruneh B.T., Beshah D.T. (2016). Medication administration error reporting and associated factors among nurses working at the University of Gondar referral hospital, Northwest Ethiopia, 2015. BMC Nurs..

[21] Červený M., Hajduchová H., Brabcová I., Chloubová I., Prokešová R., Malý J., Malá-Ládová K., Doseděl M., Tesař O., Vlček J. (2023). Self-reported medication administration errors in clinical practice of nurses: A descriptive correlation study. Med. Pr..

[22] Act of 16 June 2023 on Quality in Health Care and Patient Safety [Internet]. https://isap.sejm.gov.pl/isap.nsf/download.xsp/WDU20230001692/T/D20231692L.pdf.

[23] Pawłowska I., Kocić I. (2014). Rational use of medicines in the hospitals of Poland: Role of the pharmacists. Eur. J. Hosp. Pharm..

[24] Kwiecień-Jaguś K., Paluch K., Małecka-Dubiela A., Mędrzycka-Dąbrowska W., Kopeć M. (2022). Reporting Adverse Drug Events in Drug Administration Process among Nursing Personnel. J. Neurol. Neurosurg. Nurs..

[25] Sanghera I.S., Franklin B.D., Dhillon S. (2007). The attitudes and beliefs of healthcare professionals on the causes and reporting of medication errors in a UK Intensive care unit. Anaesthesia.

[26] LoBiondo-Wood G., Haber J. (2021). Nursing Research Methods and Critical Appraisal for Evidence-Based Practice.

[27] Ding W., Chen J., Liu J., Lin B., Li S., Li F., Guo J., Li Y., Li J. (2022). Development and validation of the Health Education Adherence Scale for Stroke Patients: A cross-sectional study. BMC Neurol..

[28] Watson R., Thompson D.R. (2006). Use of factor analysis in Journal of Advanced Nursing: Literature review. J. Adv. Nurs..

[29] Zyoud S.H., Khaled S.M., Kawasmi B.M., Habeba A.M., Hamadneh A.T., Anabosi H.H., Fadel A.B., Sweileh W.M., Awang R., Al-Jabi S.W. (2019). Knowledge about the administration and regulation of high alert medications among nurses in Palestine: A cross-sectional study. BMC Nurs..

[30] The Minister of Health Types, Scope and Patterns of Medical Documentation and the Methods of Procesing It [Internet]. Regulation of the Minister of Health. https://isap.sejm.gov.pl/isap.nsf/download.xsp/WDU20200000666/O/D20200666.pdf.

[31] Aldossary D.N., Almandeel H.K., Alzahrani J.H., Alrashidi H.O. (2022). Assessment of Medication Errors Among Anesthesia Clinicians in Saudi Arabia: A Cross-Sectional Survey Study. Glob. J. Qual. Saf. Healthc..

[32] Salvini A., Silva E., Passos C., Manuel T., Moraes C., Sousa C., Alves P. (2024). Validation of ELPO-PT: A Risk Assessment Scale for Surgical Positioning Injuries in the Portuguese Context. Nurs. Rep..

[33] Härkänen M., Vehviläinen-Julkunen K., Murrells T., Rafferty A.M., Franklin B.D. (2019). Medication administration errors and mortality: Incidents reported in England and Wales between 2007–2016. Res. Soc. Adm. Pharm..

[34] Kane-Gill S., Weber R.J. (2006). Principles and Practices of Medication Safety in the ICU. Crit. Care Clin..

[35] Institute for Safe Medication Practices High-Alert Medications in Acute Care Settings | Institute for Safe Medication Practices [Internet]. https://www.ismp.org/recommendations/high-alert-medications-acute-list.

[36] Hanna G.M., Levine W.C. (2011). Medication Safety in the Perioperative Setting. Anesthesiol. Clin..

[37] Martin L.D., Grigg E.B., Verma S., Latham G.J., Rampersad S.E., Martin L.D. (2017). Outcomes of a Failure Mode and Effects Analysis for medication errors in pediatric anesthesia. Pediatr. Anesth..

[38] Zacharowski K., Filipescu D., Pelosi P. (2023). Intensive care medicine in Europe: Perspectives from the European Society of Anaesthesiology and Intensive Care: Erratum. Eur. J. Anaesthesiol..

[39] International Medication Safety Network Existing National Reporting and Learning Systems [Internet]. https://www.intmedsafe.net/the-international-medication-error-reporting-portal/existing-national-reporting-and-learning-systems/.

[40] Cranovsky R.S., Krajewski R. Zdarzenia Niepożądane w Lecznictwie i Błędy Medyczne–skala Problemu. https://www.mp.pl/artykuly/57851,zdarzenia-niepozadane-w-lecznictwie-i-bledy-medyczne-skala-problemu.

