# Supplementary material for: Steps to Adapt the Medication Administration Error Survey in Highly Specialised Units—Polish Perspectives
Source: Nurs Rep. 2025 May 14;15(5):173. doi: 10.3390/nursrep15050173 (PMC12114384; doi:10.3390/nursrep15050173)
Supplement: Supplementary file 1 [file nursrep-15-00173-s001.zip › nursrep-3577323-supplementary.pdf]

Table S1. The Medication Administration Error Scale (MAE) – part A.

A. Reasons Why Medication Errors Occur On Your Unit. Please circle the number that best reflects the extent to which you agree that the following reasons contribute to why medication errors occur on your unit.

	Strongly Disagree	*Moderately Disagree*	*Slightly Disagree*	*Slightly Agree*	*Moderately Agree*	*Strongly agree*
1. The names of many medications are similar	1	2	3	4	5	6
2. Different medications look alike	1	2	3	4	5	6
3. The packaging of many medications is similar	1	2	3	4	5	6

Table S2. The **Medication Administration Error Scale (MAE) – part B.**

Reasons Why Medication Administration Errors Are Not Reported On Your Unit. Please circle the number that best reflects the extent to which you agree that the following reasons contribute to why errors are not reported on your unit.]

	Strongly Disagree	*Moderately Disagree*	*Slightly Disagree*	*Slightly Agree*	*Moderately Agree*	*Strongly agree*
30. Nurses do not agree with hospital's definition of a medication error	1	2	3	4	5	6
31. Nurses do not recognize an error occurred	1	2	3	4	5	6
32. Filling out an incident report for a medication error takes too much time	1	2	3	4	5	6

Table S3. The **Medication Administration Error Scale (MAE) – part C.**

C. Percentage of Each Type of Error Reported on Your Unit. Based on your experience, please circle the number that best represents what percentage of each type of medication error you believe is actually reported on your unit.

	Percentage reported									
**Types of Non-IV Medication Errors**	0 - 20	21- 30	31- 40	41 - 50	51 - 60	61 - 70	71 - 80	81 - 90	91 - 99	100
46. Wrong route of administration	1	2	3	4	5	6	7	8	9	10
47. Wrong time of administration	1	2	3	4	5	6	7	8	9	10
48. Wrong patient	1	2	3	4	5	6	7	8	9	10

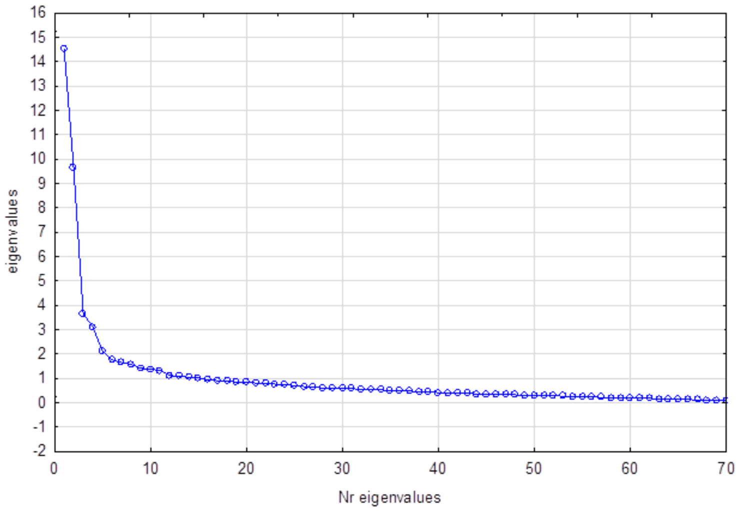

Figure S1. Scree plot
